# Microfluidic microwave biosensor based on biomimetic materials for the quantitative detection of glucose

**DOI:** 10.1038/s41598-022-20285-6

**Published:** 2022-09-24

**Authors:** Mengqi Zhang, Xiaojun Yang, Mengna Ren, Sui Mao, Rajendra Dhakal, Nam-Young Kim, Yuanyue Li, Zhao Yao

**Affiliations:** 1 College of Micro and Nano Technology, Qingdao University, Qingdao, 266071 China; 2 College of Materials Science and Engineering, Qingdao University, Qingdao, 266071 China; 3 Department of Computer Science and Engineering, Sejong University, Seoul, 05006 Korea; 4 Department of Electronic Engineering, Kwangwoon University, Seoul, 01897 Korea

**Keywords:** Electrical and electronic engineering, Pre-diabetes, Sensors and biosensors

## Abstract

This paper presents a microwave microfluidic biosensor for monitoring blood glucose levels. The glucose sensor is a triple ring microstrip patch antenna integrated with a biomimetic microfluidic device capable of measuring a fixed volume of glucose solution. The sensor was utilized to detect 50–500 mg/dL glucose solutions. The interaction of the glucose solution with the electromagnetic field on the patch's surface influences both the resonance frequency and the magnitude of reflection coefficient. The results indicate that the microfluidic device can reduce experimental error and enhance the correlation between glucose concentration, resonant frequency, and reflection coefficient. Finally, the microfluidic sensor had a sensitivity of 0.25 MHz/(mg/dL), a detection limit as low as 7.7 mg/dL, and correlation coefficients of resonance frequency and reflection coefficient with a glucose concentration of 0.996 and 0.984, respectively. The experiment on the sensor's stability verifies the sensor's excellent stability and rapid response (~ 150 ms). Consequently, the device can be used to differentiate the concentration of glucose solutions, as well as to detect blood glucose levels at an early stage.

## Introduction

According to survey data released by the International Diabetes Federation (IDF) in 2021, the global prevalence of diabetes is rising and is projected to reach 783,2 million by 2045^1^. To prevent complications such as blindness, renal failure, and cardiovascular and cerebrovascular diseases^2,3^, patients should conduct self-monitoring of blood glucose levels (BGL). Currently, the most prevalent method for detecting BGL involves drawing blood from the fingertips, which not only causes physical discomfort but also pollutes the environment with single-use test strips. Therefore, it is unsuitable for routine BGL monitoring. As a result, an urgent need exists for a high-precision, stable, low-cost, and noninvasive BGL monitoring technology.

A growing number of techniques have been used to monitor BGL in real-time, including optical^4–6^, electrochemical^7,8^, microwave^9–18^, and other techniques^19,20^. The common method of analysis in optics is near-infrared spectroscopy^21^, but there are still limitations. For instance, because biological tissue contains a great deal of water, the light attenuation is so severe that the signal is weak, affecting its precision. The most common electrochemical technique is reverse iontophoresis^22^, which extracts glucose from tissue fluid to the skin surface and then measures its concentration using an electrochemical sensor. These sensors have a short lifespan, and their precision degrades over time. Moreover, because microwave technology has the benefits of simple design and fabrication, low cost, high sensitivity, and rapid detection speed, it is increasingly used for biomedical research, including the measurement of BGL^23^. Changes in glucose concentration have been shown to affect the electromagnetic properties of blood, specifically the dielectric constant. This change is detectable by the microwave sensor due to the relationship between its frequency response and the dielectric properties of the material with which it interacts^24^. Due to the complexity of blood, the vast majority of studies in the field of microwave-assisted glucose-sensing research use aqueous glucose solutions as experimental validation test samples^25^. Vidya et al.^26^ proposed three microstrip structures consisting of a helical antenna, a narrowband antenna, and a wideband antenna as microwave sensors for non-invasive measurement of BGL, which is measured by return loss. To achieve more sensitive and accurate sample detection, some studies have combined microfluidic devices with microwave devices^27–34^. Microfluidic devices are often placed in the sensing area, which not only fixes the position of the test sample, but also requires less sample volume. Gianluca et al.^27^ proposed a rectangular microwave resonant cavity employing a Teflon capillary as a microfluidic channel for glucose concentration detection. However, this structure's detection limit is high and sensitivity is relatively low. Amyrul et al.^29^ proposed a microwave sensor based on a circular substrate integrated waveguide (CSIW) and a glass tube for measuring micro-volumes of glucose solutions that exhibit a similar lack of sensitivity. Ratnesh Kumari et al.^31^ presented an epsilon negative (ENG) unit cell resonator as a microwave sensor for characterizing life-sustaining samples such as glucose. This microwave sensor's microfluidic device is made of PDMS. This structure has an extremely low detection limit (0.04 mg/dL), but its sensitivity should be enhanced.

In the study mentioned above, the microfluidic device was composed of Teflon capillary, glass, and PDMS. Although the position and volume of the sample to be tested can be accurately fixed, the sensitivity is relatively low, and most detection limits are also high. Biomimetic microfluidics are ideally suited for simulating physiological functions at the tissue and organ levels in vitro. This paper proposes a biomimetic microfluidic device with an integrated microwave sensor for BGL monitoring. The sensor consists of a three-ring microstrip patch antenna with defective ground structures (DGSs). The DGSs influence the current distribution in the ground plane, thereby increasing the transmission line's capacitance and inductance. As the study sample in this work, an aqueous glucose solution was selected. Different concentrations of glucose solutions have distinct dielectric properties, resulting in modifications to the sensor's resonant frequency and reflection coefficient. By optimizing the resonant frequency, the sensitivity and detection limit are calculated, and the stability and response time of the sensor are also analyzed and explained.

## Design of the biosensor

### Design of biosensor based on triple ring patch antenna

**Figure 1:**
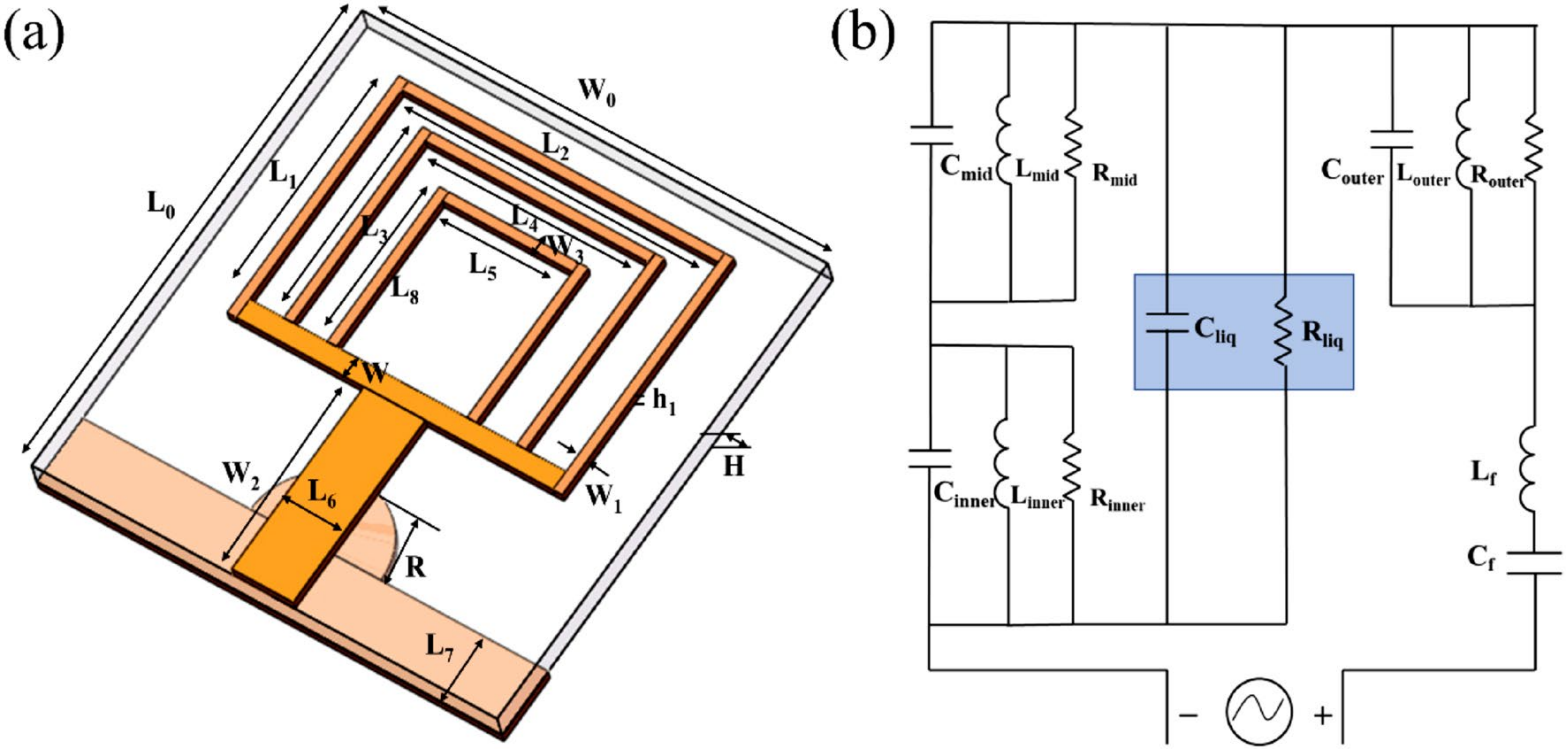
(**a**) The 3D structure of the triple ring patch antenna, (**b**) equivalent circuit model of the triple ring antenna loaded with the measured liquid.

The biosensor proposed in this paper is comprised of a triple ring microstrip patch antenna with DGS. Figure 1 depicts that the front side of the triple ring patch consists of microstrip line and triple ring structures, whereas the backside of the DGS consists of a rectangle and a semicircle structure. The dimensions of the antenna's structure are displayed in detail in Table 1. The microwave sensor was also fabricated using a Teflon (Taconic TLX-8) substrate with a dielectric constant of 2.55, a loss tangent of 0.0019, and a thickness of 0.508 mm. The antenna structure is modeled and optimized using full-wave electromagnetic software computer simulation technology. Advanced Design System (ADS) is used to optimize the equivalent circuit of the antenna optimization procedure. The capacitance and inductance of the sample under test are denoted by *C*_*liq*_ and *R*_*liq*_ in Fig. 1b. *C*_*liq*_ and *R*_*liq*_ were connected in parallel with the RLC circuits of the inner and middle rings because the testing samples were positioned in the middle of the inner and middle rings.

**Table 1:** Detailed dimensions of the structure.

Dimension	*W*_*0*_	*W*	*W*_*1*_	*W*_*2*_	*W*_*3*_	*L*_*0*_	*L*_*1*_	*L*_*2*_
Unit (mm)	25	1	0.5	3.3	1	25	13	17

Dimension	*L*_*3*_	*L*_*4*_	*L*_*5*_	*L*_*6*_	*L*_*7*_	*L*_*8*_	*R*	
Unit (mm)	10.5	12	7	10	3.5	8.5	3.5	

### The optimization process of biosensor

**Figure 2:**
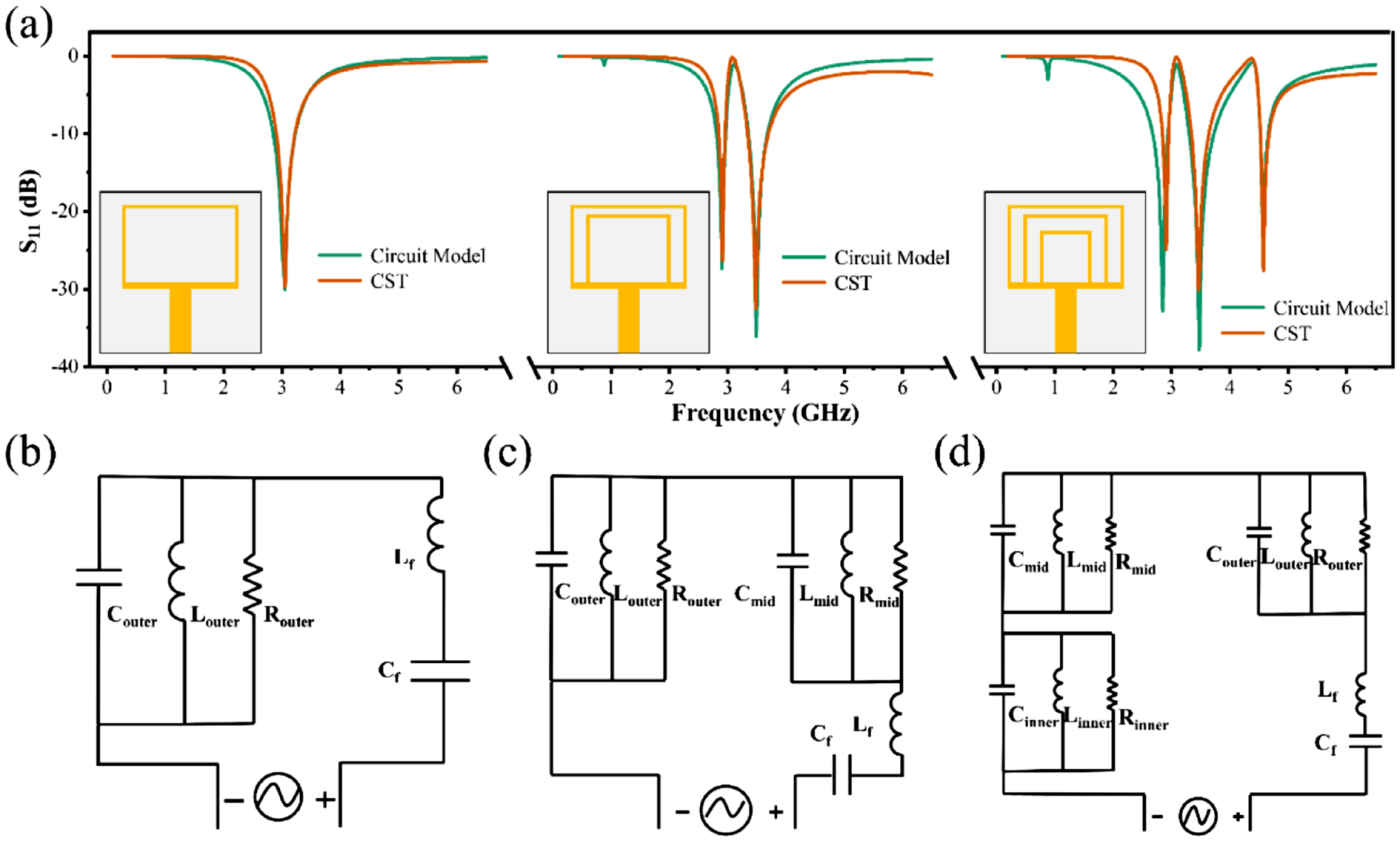
(**a**) Optimization process of triple ring antenna. Equivalent circuit model of (**b**) single ring antenna, (**c**) double ring antenna, and (**d**) triple ring antenna.

Figure 2 depicts the process of optimizing the ring antenna, including simulation results of reflection coefficient (*S*_*11*_) and an equivalent circuit model. As depicted in Fig. 2a, the single ring antenna has a single resonance at 3.04 GHz while the defect structure remains unchanged. Adding second ring results in two resonances at 2.91 GHz and 3.48 GHz, respectively. Finally, another ring is added to create a tri-band antenna with resonance points at 2.91 GHz, 3.46 GHz, and 4.58 GHz, respectively.

The equivalent circuit models of the single, double, and triple ring antenna are depicted in Fig. 2b–d, respectively. *C*_*outer*_, *R*_*outer*_, and *L*_*outer*_ represent the capacitance, resistance, and inductance, respectively, of the outer ring. *C*_*mid*_, *R*_*mid*_, *L*_*mid*_, *C*_*inner*_, *R*_*inner*_, and *L*_*inner*_ are the equivalent capacitance, resistance, and inductance of the middle and inner rings, respectively. The parameter values for the equivalent circuit model are shown in Table 2.

**Table 2:** The parameter values in the equivalent circuit model.

	L_f_ (nH)	C_f_ (pF)	R_outer_ (Ohm)	L_outer_ (nH)	C_outer_ (pF)	R_mid_ (Ohm)	L_mid_ (nH)	C_mid_ (pF)	R_inner_ (Ohm)	L_inner_ (nH)	C_inner_ (pF)
Single ring	14.043	0.1962	749	5.77	5.3						
Double ring	11.2	0.221	749	5.788	5.395	730.9	0.375	7.018			
Triple ring	6.51	0.38	749	5.77	5.24	730.9	0.3012	8.84	950	0.2124	6.14

**Figure 3:**
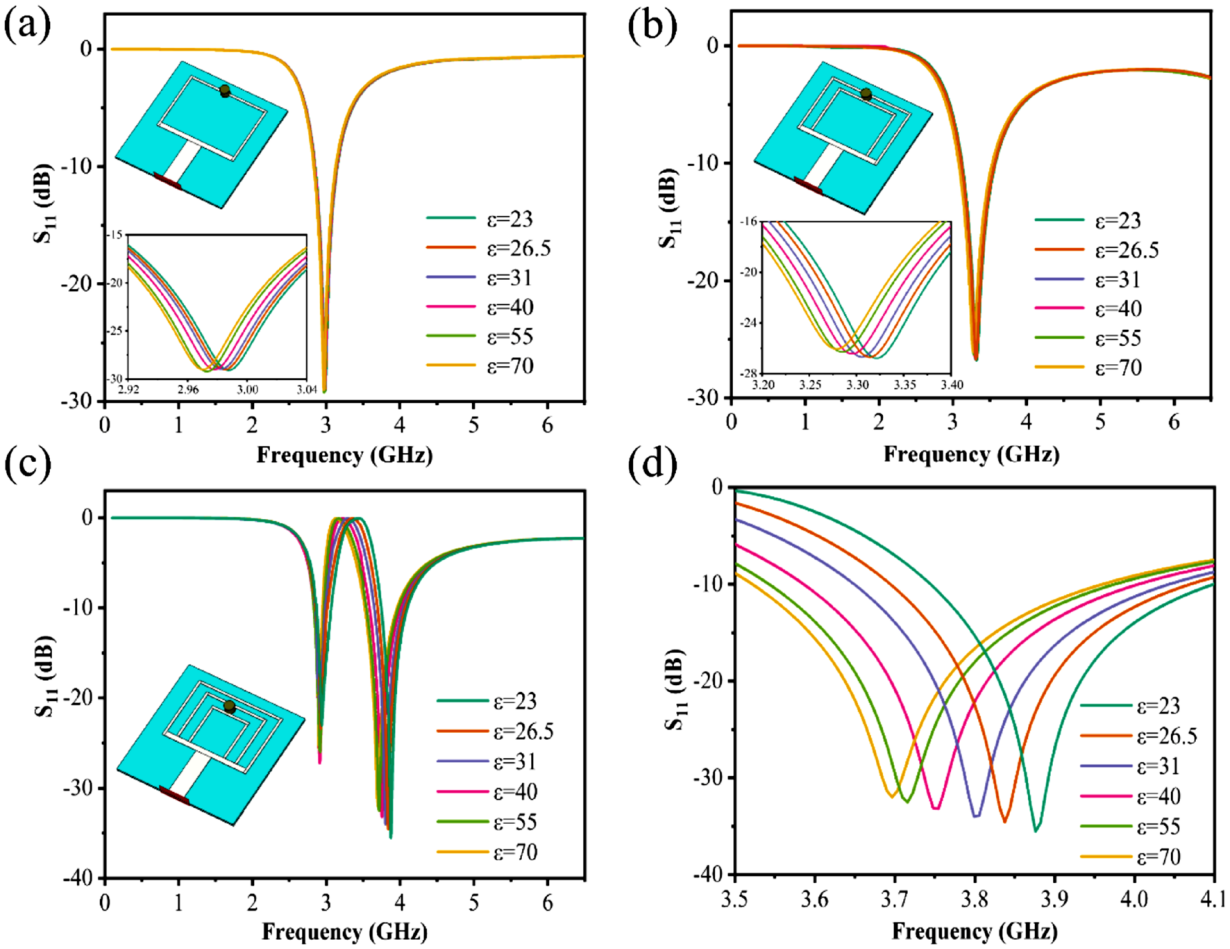
(**a**) Simulation results of a single ring antenna for samples with different dielectric constants (the inset shows the location of the test), (**b**) simulation results of a double ring antenna for samples with different dielectric constants, (**c**) triple ring antennas for different dielectric constants simulation results of the sample, (**d**) enlarged view of the results of the triple ring antenna simulation test at 3.5–4.1 GHz.

In order to illustrate the principle of triple ring antenna was chosen, the results of three proposed structures were simulated. Figure 3a shows the variation of the dielectric constant from 23 to 70 for the simulation sample of the single ring antenna, and the inset depicts the location of the test sample. As the sample's dielectric constant increases, the sample's resonant frequency shifts to a lower frequency. Figure 3b depicts the double ring antenna simulation test result. Since the sample is on both rings at once, the addition of the sample causes the two resonances to merge into one. And as the sample's dielectric constant decreases, the resonant frequency rises. Figure 3c depicts the simulation result for the triple ring antenna, while Fig. 3d depicts an enlarged view at 3.5–4.1 GHz. This structure positions the test sample on the middle and inner ring, so that when the sample is dropped, the first resonance has little effect, and the second and third resonances combine into one. As the dielectric constant increases, the resonant frequency also decreases. The sensitivity then increases significantly to the final triple ring as the number of rings increases. To achieve a high sensor sensitivity level, the triple-ring patch antenna with a wide range of variation is chosen in this study.

**Figure 4:**
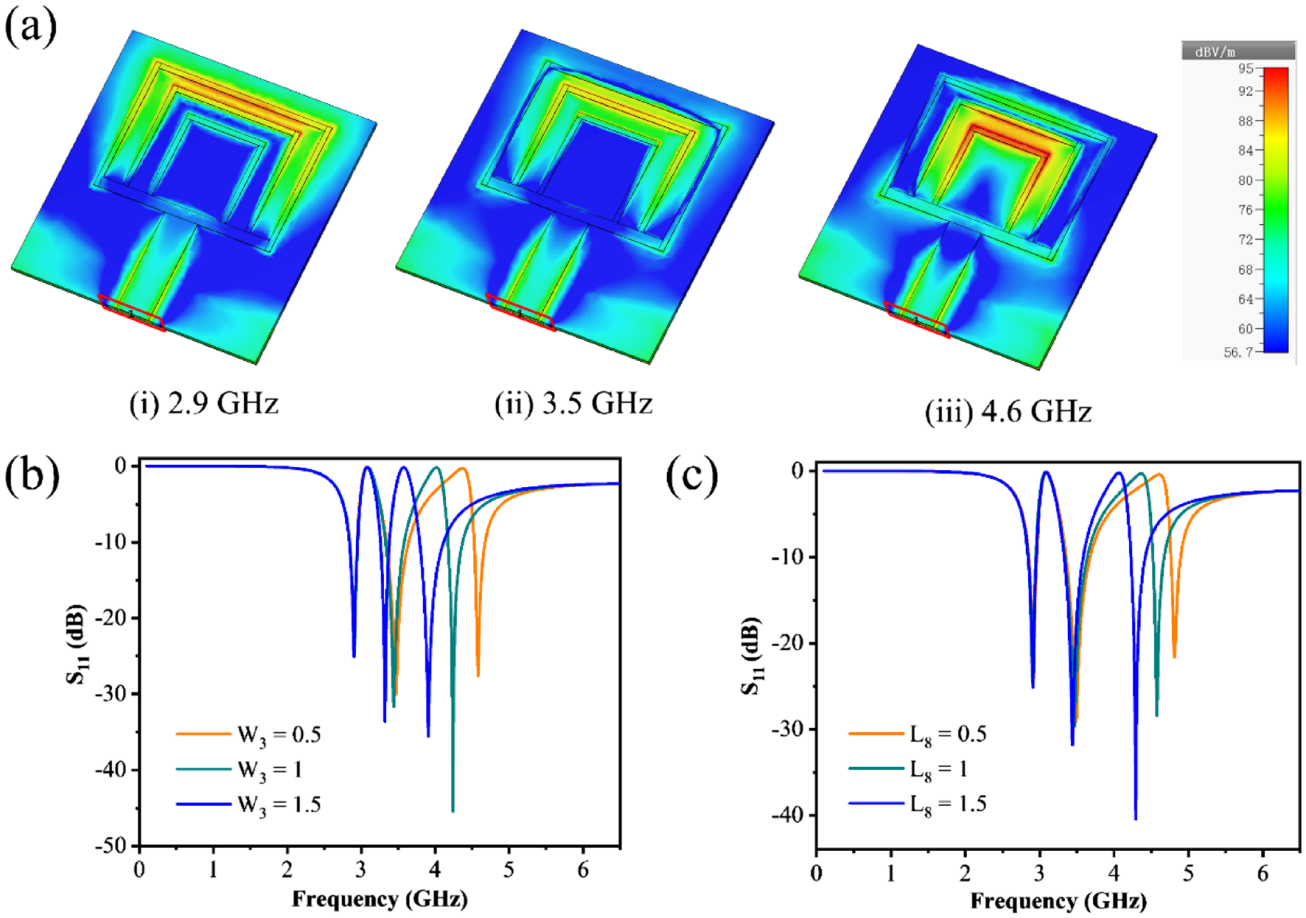
(**a**) Electric field distribution of the triple ring antenna at different resonant frequencies, and simulated responses of the proposed triple ring antenna for different values (**b**) the width of *W*_*3*_, (**c**) the height of *L*_*8*_.

Figure 4a depicts the repetitive electric field distribution of the triple ring antenna at 2.9 GHz, 3.5 GHz, and 4.6 GHz, respectively. It can be seen that the electric field is concentrated in the center of the outer ring and the middle ring at 2.9 GHz. At the resonance frequency of 3.5 GHz, the electric field is concentrated around the middle ring. However, at 4.6 GHz, the electric field distribution is most concentrated in the center of inner ring and middle rings. As a result, the positions of the inner ring and the middle ring are chosen as the sensing area for a more sensitive sensor.

The influence of different parameter values is also investigated to obtain a structure with improved performance. Figure 4b illustrates the effect of *W*_*3*_ on the *S*_*11*_ for the inner ring. It can be observed that the first resonance remains unchanged as *W*_*3*_ increases. However, the second and third resonances are shifted to the left part, with the third resonance being shifted more. In conjunction with the fluctuating amplitude of *S*_*11*_, *W*_*3*_ is finally fixed as 1 mm. Figure 4c illustrates the effect of the inner ring's height (*L*_*8*_). The results demonstrate that the modification of *L*_*8*_ has almost no effect on the first and second resonances. However, it can only influence the third resonance. When *L*_*8*_ is decreased, it will be shifted to higher frequency, and the value of *S*_*11*_ will be decreased. As a result, the affinity of the key parameters coincides precisely with the distribution of the electric field.

## Results

The single, double, and triple sensors were fabricated and tested, respectively. Figure S1 shows the test results of single and double sensors. Since the maximum resolution of the VNA used is 4 MHz, and the sensitivity of the single ring sensor is lower than 4 MHz, the variation of the performance parameters with the concentration of the glucose solution cannot be detected, as shown in Fig. S3a. Figure S3b shows the test results of the double ring sensor. Compared with the results of the triple ring sensor, the sensitivity of the double ring sensor is significantly smaller, so the subsequent measurements are made of the triple ring sensor.

**Figure 5:**
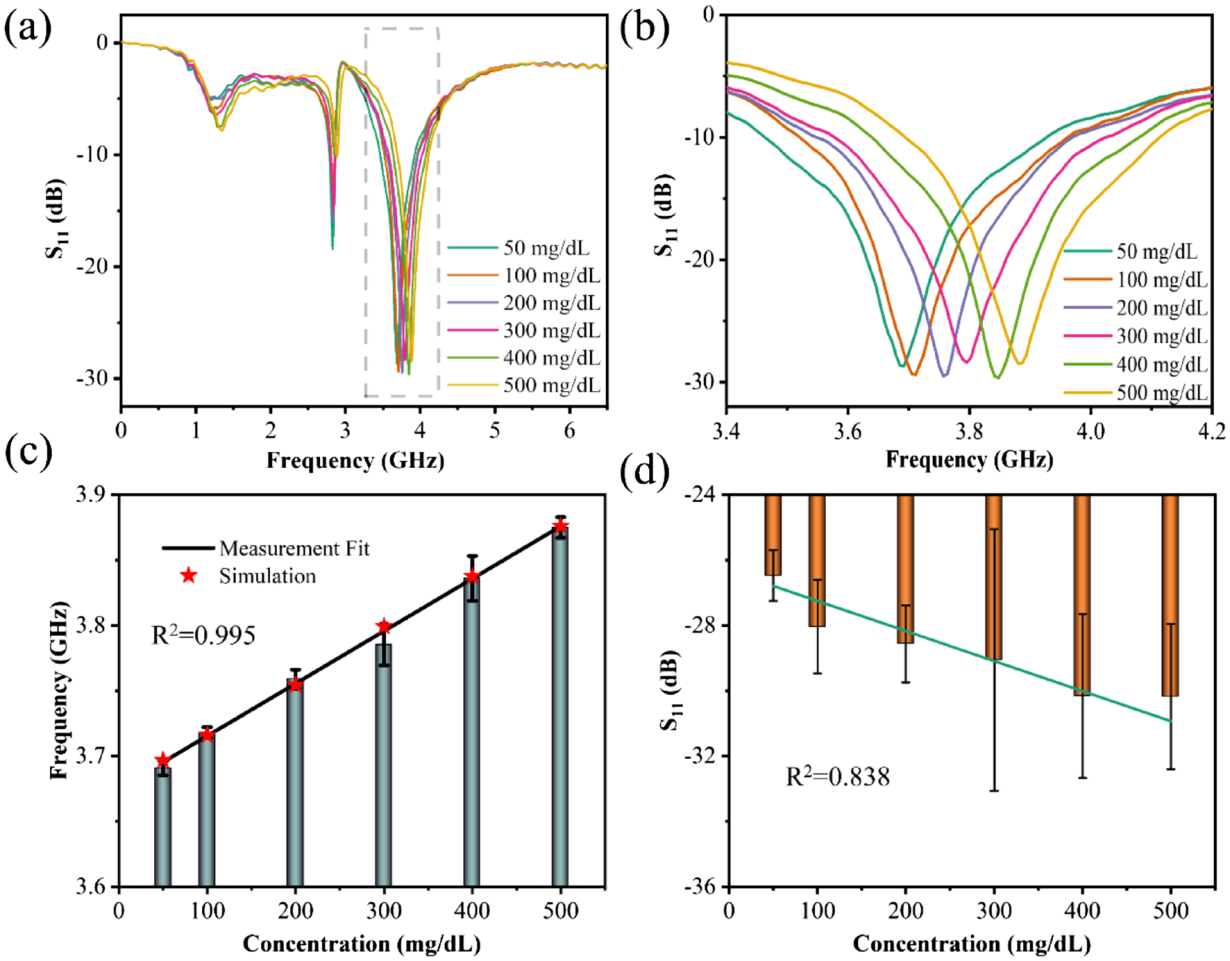
The measurement results of the proposed sensor for glucose solution in the concentration range of 50–500 mg/dL, (**a**) the overall change diagram, (**b**) the enlarged view of 3.4–4.2 GHz, (**c**) the linear fitting diagram of the resonance frequency with the concentration of glucose solution, (**d**) the linear fitting diagram of *S*_*11*_ with the variation of the concentrations.

Figure 5 shows the measurement results of the triple ring sensor for glucose solutions in the concentration range of 50–500 mg/dL. Figure 5a is the overall RF results from 0.1 to 6.5 GHz, and Fig. 5b is an enlarged view in the range of 3.4 to 4.2 GHz. As the concentration of the glucose solution increases, the resonant frequency increases accordingly. And from the results of multiple measurements, its *S*_*11*_ decreases accordingly. Figure 5c,d are the linear fittings of the resonance frequency and *S*_*11*_ with different concentrations of glucose solution, respectively. The resonance frequency increases linearly with the increase of the solution concentration, and the correlation coefficient (*R*^2^) is 0.995. The star shape in the figure is the simulation result, which shows excellent agreement with measured results. The value of *S*_*11*_ also decreases linearly with the increase of solution concentration, but its error is a little larger, and the *R*^2^ is only 0.838.

**Figure 6:**
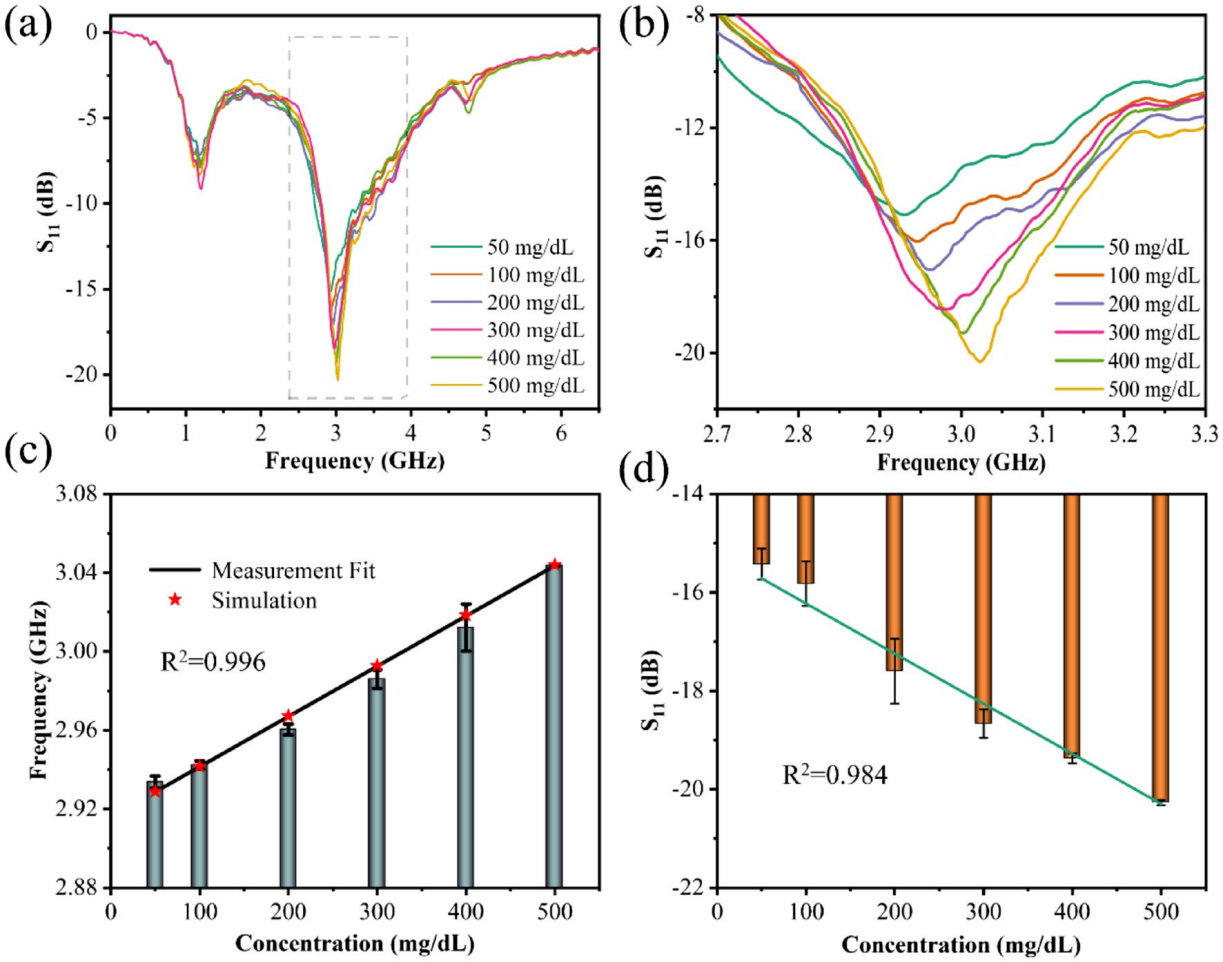
Measurement results of the sensor based on the microfluidic device for glucose solutions in the concentration range of 50–500 mg/dL. (**a**) The overall change in 0.1–6.5 GHz, (**b**) enlarged view in 2.7–3.3 GHz, (**c**) the linear fitting diagram of the resonance frequency with the concentration of glucose solution, (**d**) the linear fitting diagram of *S*_*11*_ with the variation of the concentration of glucose solution.

A microfluidic device was integrated with the microwave sensor to reduce experimental measurement error and improve the correlation between performance parameters and solution concentration. The integrated devices are utilized for testing to improve the structure's measurement results. Likewise, Fig. 6a represents the overall measurement result. Due to the integration of the microfluidic device into the device, its resonant frequency shifts to 3.1 GHz in the absence of glucose solution. Figure 6b is a magnified view of the range between 2.7 and 3.3 GHz. The resonance frequency shifts to the right as the concentration of glucose solution increases, while *S*_*11*_ decreases significantly. Figure 6c,d are the fitting diagrams of the resonant frequency and the relationship between solution concentration. The resonance frequency increases linearly with the concentration of the solution, the error decreases slightly, and *R*^2^ can reach 0.996. In addition, the variation of *S*_*11*_ with the concentration of the solution is drastically improved, with *R*^2^ increasing to 0.984 compared to the microwave sensor without a microfluidic device.

**Figure 7:**
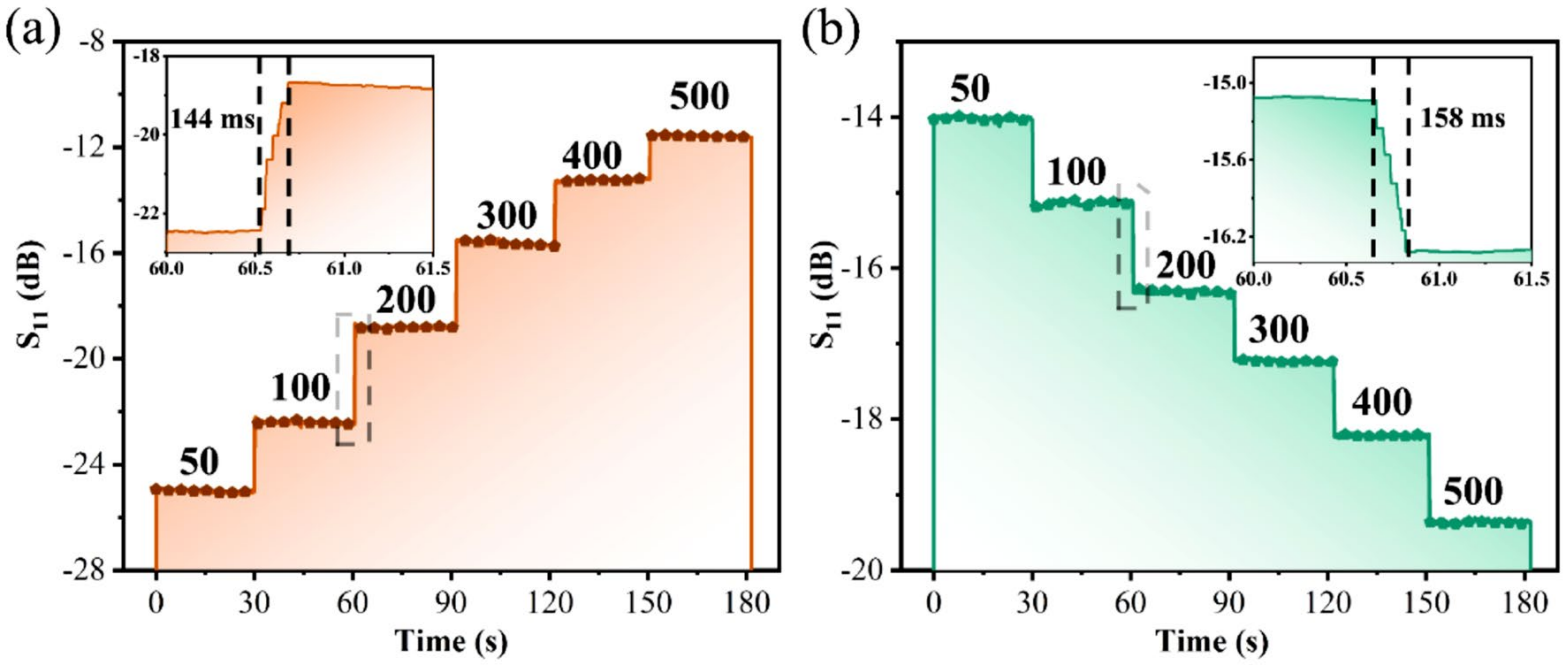
(**a**) The stability test results in the concentration range of 50–500 mg/dL for (**a**) bare biosensor chip and (**b**) the biosensor combined with the microfluidic device (the insets show the response time of the different solution concentration).

Following that, the sensor's stability was examined. Figure 7a,b show the stability results of the bare sensor and the sensor integrated with the microfluidic device, respectively. The results demonstrate that both sensors exhibit good stability over a certain time period. In the meantime, the insets depict the response time for the sensor-only and sensor-integrated microfluidic devices, with response times of 144 ms and 158 ms, respectively. As a result, Table 3 compares the proposed sensor to other microfluidic sensor literature. The sensitivity in the Table 3 is defined as the offset of the resonance frequency corresponding to the concentration per mg/dL, the formula is *S* = Δ*f*/Δ*c*, Δ*f* is the frequency value of the offset, and Δ*c* is the value of the changed concentration. The detection of limit (*lod*) is the minimum concentration of the substance to be tested that can be detected from the sample. The formula is lod = *k*·*S*_*b*_/*m*, where *k* is a constant related to the confidence concentration, take *k* = 3, *S*_*b*_ is the blank standard deviation, and *m* is the slope of the analytical calibration curve in the low concentration range. Evidently, the proposed sensor has a relatively low detection limit and high sensitivity.

**Table 3:** Comparison of the proposed microfluidic sensor and other microfluidic sensors (the test results of glucose solution are used for the comparison of sensitivity). *N.A.* Not Available, – the sensor without a microfluidic device.

References	Technique	Microfluidic material	LOD (mg/dL)	Sensitivity (MHz/(mg/dL))	Response time
^14^	Loss-compensated split-ring resonator (SRR)	–	18	1.33E−05	< 1 s
^15^	Split-ring resonator (SRR)	–	600	1.64E−04	30 min
^18^	Patch antenna	–	N.A.	2.575E−04	< 1 s
^27^	Rectangular waveguide cavity	Teflon capillary	260/420	4.00E−04	N.A.
^29^	Circular CSIW resonator	Glass capillary	N.A.	3.83E−03	N.A.
^30^	Microstrip transmission line loaded with a complementary split-ring resonator (CSRR)	PDMS	N.A.	5.00E−03	N.A.
^31^	ENG unit-cell resonator	PDMS	0.04	1.00E−02	N.A.
^32^	Distributed MEMS transmission lines (DMTL)	Silicon oxide	N.A.	1.64E−02	N.A.
^16^	Interdigitated capacitor (IDC) resonator-etched coplanar waveguide (CPW)	–	50	2.00E−02	N.A.
^34^	Complementary electric-LC resonator coupled with a microstrip line	PDMS	N.A.	2.11E−02	N.A.
^13^	Spoof Fano resonance structure	–	10	5.4E−02	N.A.
This work	Patch antenna	Biomimetic materials	7.7	2.45E−01	158 ms

## Discussion

The microwave sensor's detection principle is based on the change in the sample's dielectric properties. According to the Debye dispersion model, the dielectric constant ($\varepsilon^{\prime}_{{\text{g}}}$) and loss factor ($\varepsilon^{{\prime\prime}}_{{\text{g}}}$) of the glucose sample can be expressed as complex numbers as follows^35,36^:

$$ \varepsilon_{g} = \varepsilon^{{\prime}}_{g} + j\varepsilon^{{\prime\prime}}_{g} = \left[ {\frac{{\left( {\varepsilon_{s} - \varepsilon_{\infty } } \right)}}{{1 + \omega^{2} \tau^{2} }} + \varepsilon_{\infty } } \right] + j\left[ {\frac{{\left( {\varepsilon_{s} - \varepsilon_{\infty } } \right)\omega \tau }}{{1 + \omega^{2} \tau^{2} }}} \right] $$

The above equation is an approximation to study the effect of glucose concentration on the dielectric constant of the sample. Monosaccharide molecules (C_6_H_12_O_6_) contain more –OH groups. When these groups are present in water as monomers, more –H bonds are formed, resulting in less water available to interact with the AC field^37^. Therefore, the dielectric constant of the water-glucose solution is lower than that of water, and the dielectric constant decreases with increasing glucose concentration. Figure 9d is a schematic diagram of the proposed glucose sensor. Figure 9b,c are the SEM images of the bare sensor and the measured sensor, respectively. The surface of the device has some changes before and after glucose solution measurement. Figure S1 shows the simulation and measurement comparison of the optimized sensor, which shows good consistency.

Finally, the limitations of the biosensor and the future research are discussed. The biosensor can be optimized for both miniaturization and further sensitivity improvement. The size of the biosensor is 25 × 25 mm^2^, which has been reduced compared to previous studies. But in order to obtain a more compact and portable integrated system, the size of the device should be further reduced. To solve this problem, air bridge capacitors can be designed and fabricated on a gallium arsenide (GaAs) substrate using an integrated passive device (IPD) technology. The air bridge structure is introduced into the external metal wire to improve the capacitance per unit volume, which can achieve high sensitivity and miniaturization of the device. Improving the sensitivity of biosensors is a systematic work. Firstly, the sensitive area of the sensor needs to be considered, and the simulation software is used to determine the current intensity and whether the sensitive area is sensitive enough. Also, the defected ground structures (DGS) can be considered, which has a relatively good advantage in improving the sensitivity. Secondly, the sensitivity can be improved by adding an active feedback circuit. The addition of the feedback circuit can compensate for the loss and greatly improve the Q factor. By optimizing the parameters of the feedback circuit to achieve good results. Furthermore, it can be considered to use antigen–antibody for specific recognition, which can better recognize the target. By combining the above methods, the sensitivity can be well improved. The direction of future research will be based on the integrated test system. For example, by using FPGA or Single-Chip Microcomputer to realize the integration of the test system, the test can be more compact and portable.

## Conclusion

In this paper, a biomimetic microfluidic device that is integrated with a microstrip antenna to form a microfluidic-based microwave biosensor is proposed. The proposed sensor is inexpensive, simple to fabricate, and sensitive enough to meet glucose biosensing requirements. The glucose solution measurement results validate the performance of the proposed sensor. The correlation between glucose concentration, resonance frequency, and *S*_*11*_ can be enhanced by integrating a microfluidic device. The sensor's stability test validates the structure's high stability and rapid response. These results may demonstrate the viability of this structure as a glucose sensor, making it a possible candidate for monitoring blood glucose levels in patients with early-stage diabetes.

## Methods

### Fabrication of microwave sensor

**Figure 8:**
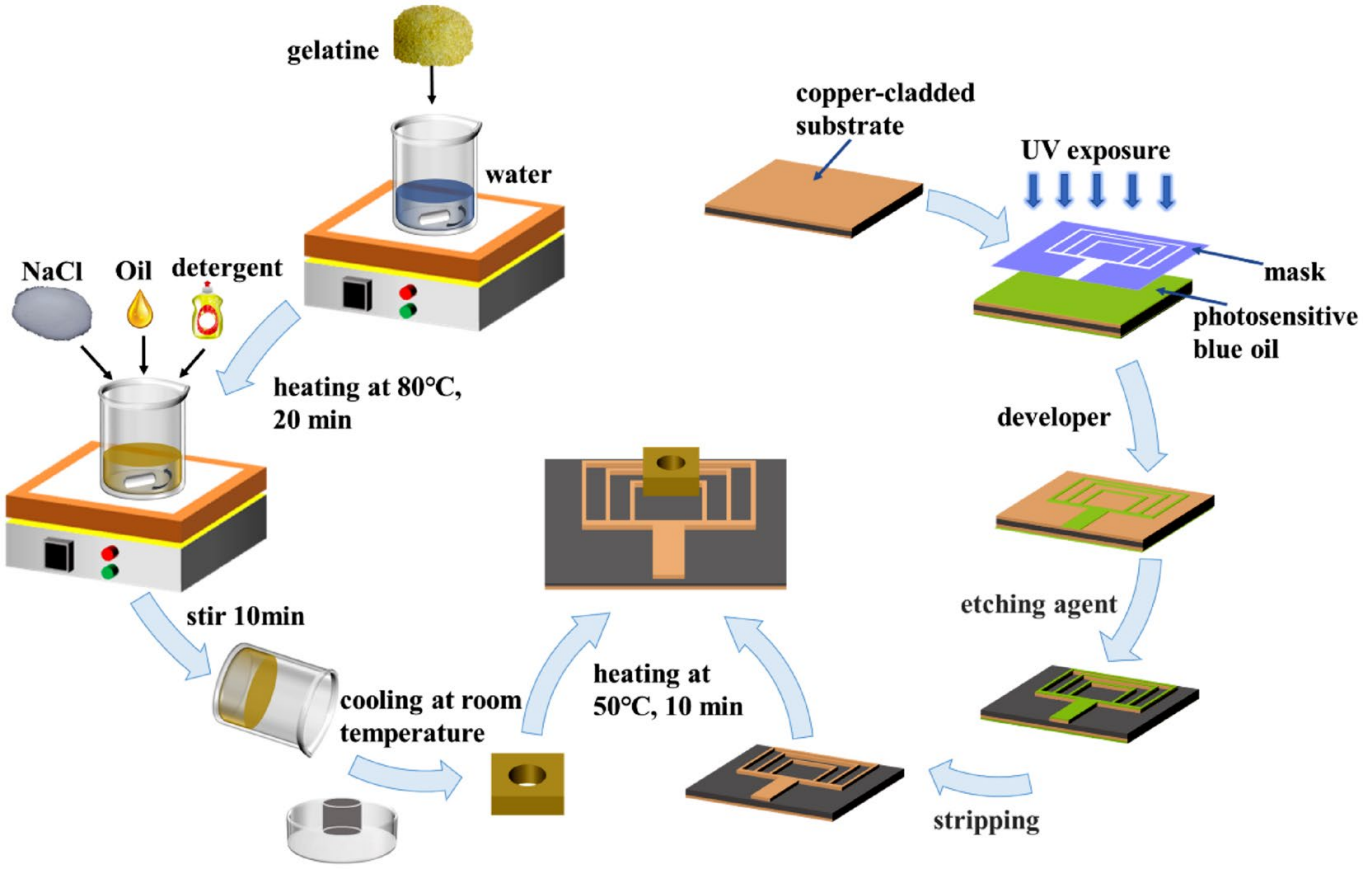
Illustration of the processes involved in the microfluidic sensor fabrication.

**Figure 9:**
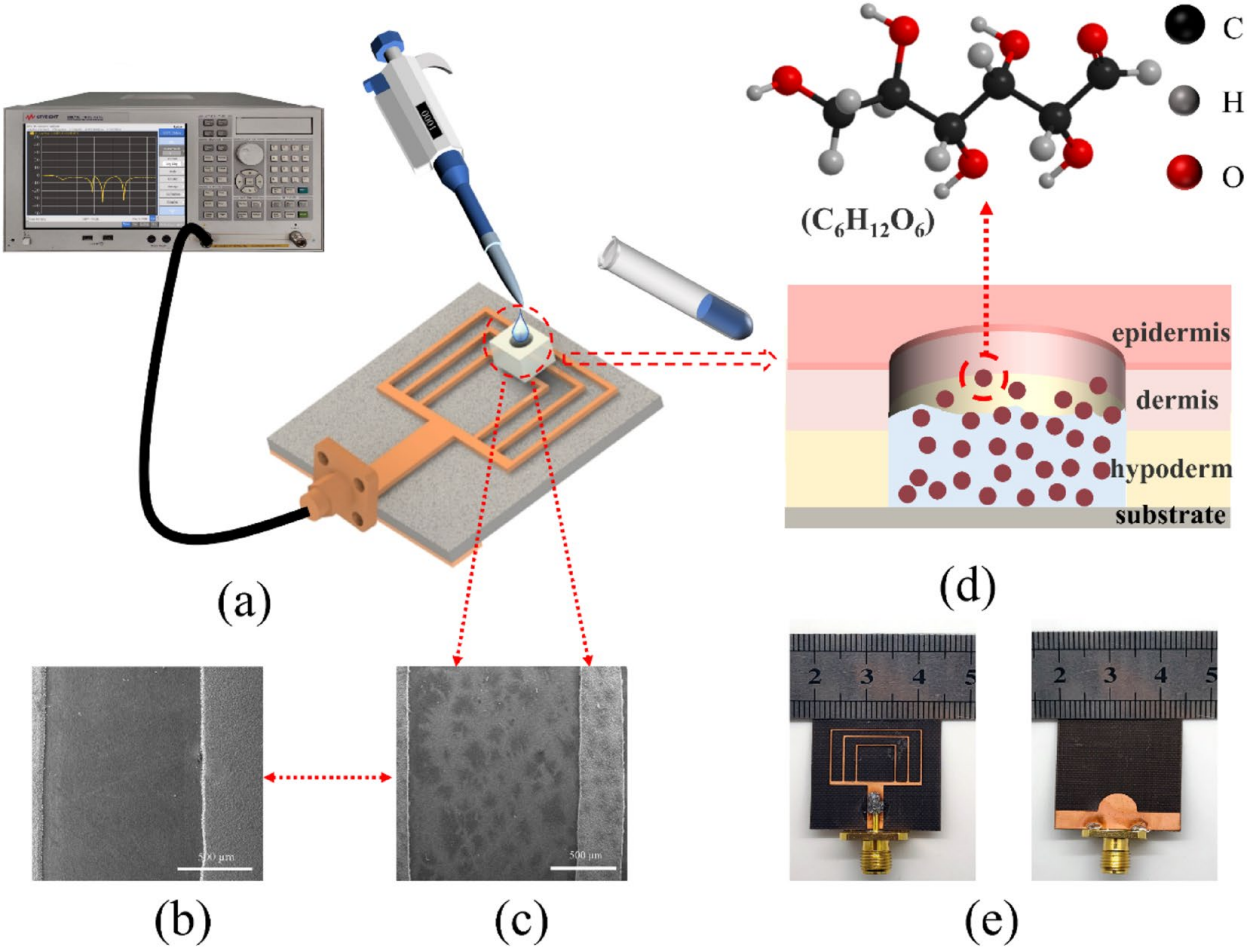
(**a**) The measurement setup for the experiment, (**b**) SEM image of the bare biosensor, (**c**) SEM image after adding the sample for measurement, (**d**) schematic diagram of the glucose biosensor, (**e**) optical photograph of the proposed biosensor (front view and back view).

The fabrication process employed to realize the proposed sensor is shown on the right side of Fig. 8. First, a uniform coating of diluted photosensitive blue oil is applied to the surface of PCB. Then, it was then heated and dried at 85 °C for 10 min on a heating plate. After the blue oil has dried, the antenna-shaped photo mask was apply to the treated PCB surface and expose it to UV light for two minutes. When the irradiation is complete, the PCB is immersed for 30 s in the developer solution. The PCB was subsequently cleaned and etched in etchant. Finally, the required microwave sensor is extracted from the blue oil. Figure 9e depicts the front and back views of the manufactured sensor.

### Fabrication of microfluidic devices

Biomimetic materials are used to fabricate the microfluidic device^38–40^. The left side of Fig. 8 shows the fabrication process of the device. The steps are show as follows: First, 34 g gelatin and 100 g water are mixed in a beaker. The beaker was heat to 80 °C in a drying oven. After the gelatin has completely melted, continue stirring and cool to 35 °C. Then, the remaining 130 g water and 1.2 g of sodium chloride were add into mixture. At room temperature, detergent and oil was combined and slowly stirred until the mixture is homogenous. Next, the homogenous mixture was poured into a container, which contains the flow channel model. Finally, the mixture was solidified to create the microfluidic device.

### Assembling of microfluidic and microwave biosensor

The microwave sensor and microfluidic device were heated for 10 min at 50 °C. The two components were then stacked and cooled to room temperature to produce a microwave sensor with a microfluidic device. Figure 8 depicts the final model in the middle of the diagram.

### Material and measurement setup

To obtain the measurement samples, the glucose powder was diluted with DI water to yield measurement samples with concentrations ranging from 50 to 500 mg/dL. Figure 9a depicts the experimental measurement setup. Vector network analyzer (VNA) was utilized to determine the *S*_*11*_ of biosensor. Through the offset of the resonant frequency and the amplitude of *S*_*11*_, the relationship between the concentration of the solution and *S*_*11*_ can be obtained. The procedure for measurement is as follows: First, use a pipette to take 2 µl of glucose solution and drop it on the sensitive area with strong electric field distribution and save the measured data. The device was then wiped clean, and the test was conducted five times. Before measuring each new set of samples, the sensor must be cleaned and dried with lint-free paper to ensure sensor repeatability.

In addition, to study the stability of the sensor, the automatic measurement program developed by the LabVIEW integrated measurement environment is used in this study. After setting the initial measurement parameters, such as scan frequency and scan interval, the program will automatically save the RF response of the microwave biosensor. By processing the measured data, the sensor's stability curve can be determined. In addition, by altering the solution's concentration, its shifting curve can correspond to its real-time response.

## Supplementary Information

Supplementary Information.

## Data Availability

The datasets used and/or analyzed during the current study available from the corresponding author on reasonable request. All data generated or analyzed during this study are included in this published article and its supplementary information files.
