# Supplementary material for: Microfluidic microwave biosensor based on biomimetic materials for the quantitative detection of glucose
Source: Sci Rep. 2022 Sep 24;12:15961. doi: 10.1038/s41598-022-20285-6 (PMC9509396; doi:10.1038/s41598-022-20285-6)
Supplement: Supplementary file 1 — Supplementary Information. [file 41598_2022_20285_MOESM1_ESM.docx]

**Supporting Information**

**Microfluidic Microwave Biosensor based on Biomimetic Materials for the Quantitative Detection of Glucose**

# Mengqi Zhang1, Xiaojun Yang1, Mengna Ren1, Sui Mao2, Rajendra Dhakal3, Nam-Young Kim4, Yuanyue Li1,*, Zhao Yao1,*

^1^Qingdao University, College of Micro & Nano Technology, Qingdao 266071, China,

^2^College of Materials Science and Engineering, Qingdao University, Qingdao 266071, China,

^3^Department of Computer Science and Engineering, Sejong University, Seoul 05006, Korea

^4^Department of Electronic Engineering, Kwangwoon University, Seoul 01897, Korea

# The simulated and measured results of the optimized sensor

The simulated and measured RF results of the optimized sensor are shown in Fig. S1. The simulated results of the sensor show three resonances at 2.91 GHz, 3.46 GHz, and 4.58 GHz, respectively. The measured results show three resonances at 2.88 GHz, 3.46 GHz, and 4.67 GHz, respectively. And the mismatching of *S_11_* at each resonance frequency is relatively small, so that the measured results showed a good agreement with simulated one.


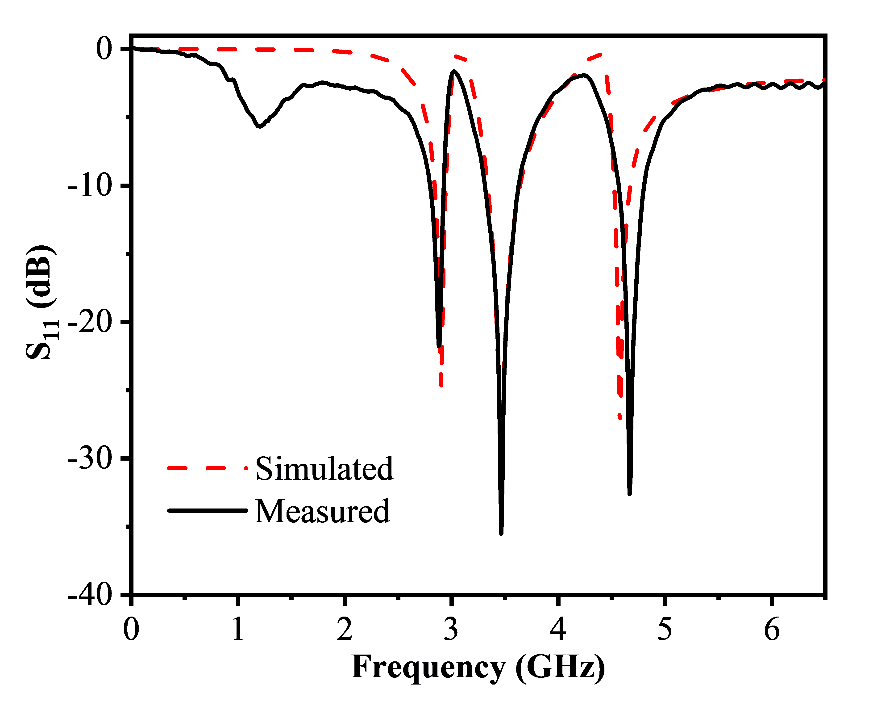


**Figure S1.** The simulated and measured results for the optimized sensor.

# Sensitivity analysis of the single ring and double ring sensors


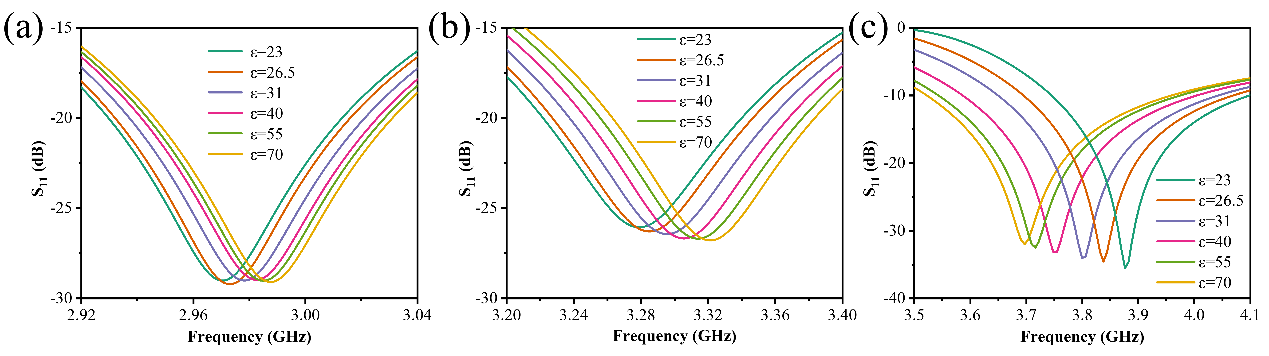


**Figure S2.** Enlarged view of the simulation results of the (a) single ring (b) double ring (c) triple ring antenna for samples with different dielectric constants

Figure S2 is an enlarged view of the comparison of the simulation test results of single ring, double ring and triple ring sensors for dielectric constants from 23 to 70. It can be concluded from the figure that when the dielectric constant changes the same, the resonant frequency of the triple ring sensor changes much more and show higher sensitivity.

Using the experimental setup of Fig. 5 (a), the single ring and double ring sensors were tested separately. Six PBS-Glucose in the range of 50 - 500 mg/dL were analyzed. Use a pipette to take 2 μL of the tested sample, drop it on the sensitive area of the sensor, and repeat the experiment 5 times to reduce the measurement error. The measured results of single ring and double ring sensors are shown in Fig. S2.

Since the resolution of the VNA is 4 MHz, and the sensitivity of the single ring sensor is lower than 4 MHz. So, the variation of the glucose concentration cannot be detected by single ring sensor, as shown in Fig. S2 (a). Figure S2 (b) is the measured results of the double ring sensor for glucose solution. Since the sensitivity of the double ring sensor is greater than 4 MHz, the changes in performance parameters with glucose concentration can be detected. As shown in the Fig. S2, when the glucose concentration increases, the resonant frequency also increases. And conversely, the amplitude of *S_11_* decreases with the increase of the glucose concentration.


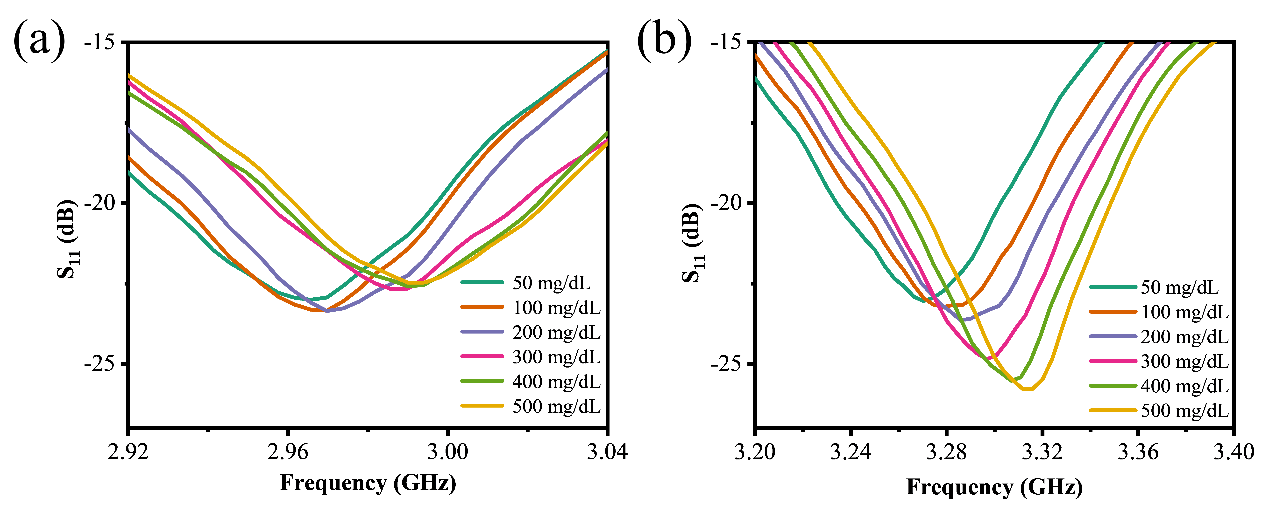


**Figure S3.** The measured results of (a) the single ring and (b) the double ring sensor for glucose solution in the concentration range of 50-500 mg/dL.

To obtain the element values in the equivalent circuit, the ADS software was used, and Fig. S4 is the overall setting diagram. The right side of Fig. S4 shows the overall composition and target settings. The *S_11_* obtained by the simulation is imported into the TB1 circuit, and the TB2 is the equivalent circuit of the structure. By setting Goal_S_11_=dB(TB2.S_11_-TB1.S_11_), the desired goal is that *S_11_* in TB2 is equal to *S_11_* in TB1, that is, Goal_S_11_ is equal to 0. Determine the approximate range of parameters through Optimize in the software, and then perform more specific optimization of parameters in Tune Parameters to achieve a better fit of the curve. Finally, the determined parameter values can be obtained.


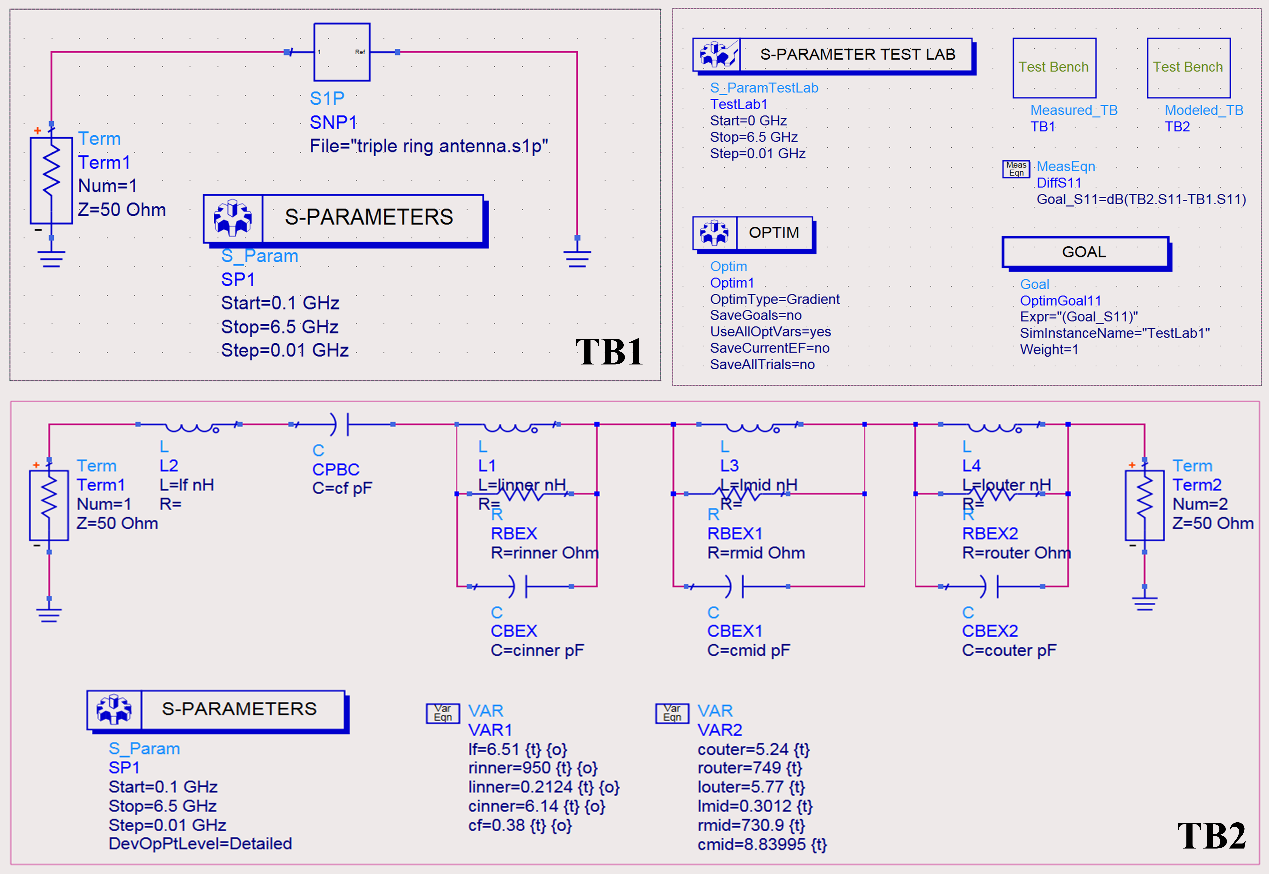


**Figure S4.** The overall setting diagram of the equivalent circuit parameter extraction using ADS.


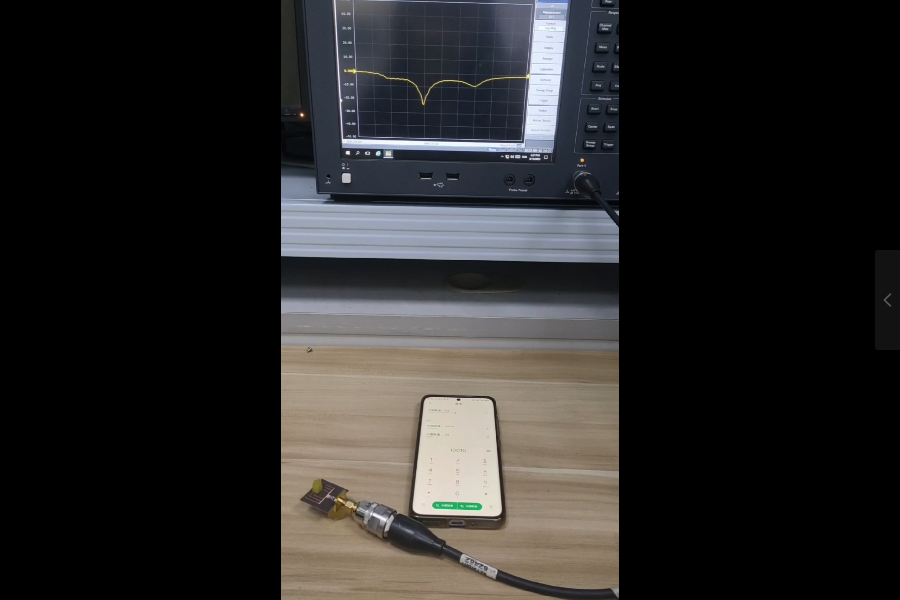


**Figure S5.** Interference test of the proposed sensor by mobile phone. (Screenshot of the Supplementary video)

To more clearly compare the performance with other literatures, Fig. S6 shows the performance comparison. The abscissa in the figure is the limit of detection, and the ordinate is the sensitivity. These two parameters are common performance representations of biosensors. The two ‘This Work’ in the figure are the measurement results with and without the microfluidic device. Figure S6 clearly shows that the proposed sensor has high sensitivity, low detection limit and fast response time.


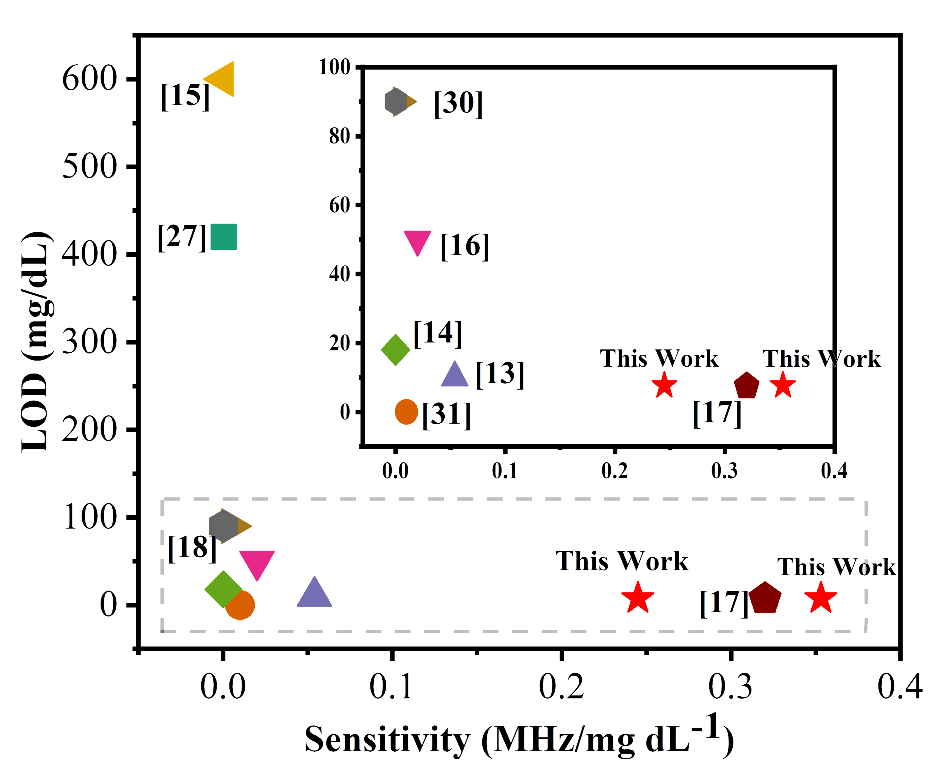


**Figure S6.** Performance comparison with other literatures.
